# Multi-Objectives Optimization of Plastic Injection Molding Process Parameters Based on Numerical DNN-GA-MCS Strategy

**DOI:** 10.3390/polym16162247

**Published:** 2024-08-07

**Authors:** Feng Guo, Dosuck Han, Naksoo Kim

**Affiliations:** Department of Mechanical Engineering, Sogang University, Seoul 04107, Republic of Korea; guof@sogang.ac.kr (F.G.); dshan@sogang.ac.kr (D.H.)

**Keywords:** plastic injection molding (PIM), multi-objective optimization, surrogate model methodologies, carbon fiber-reinforced polymers (CFRPs), multiple structural performance

## Abstract

An intelligent optimization technique has been presented to enhance the multiple structural performance of PA6-20CF carbon fiber-reinforced polymer (CFRP) plastic injection molding (PIM) products. This approach integrates a deep neural network (DNN), Non-dominated Sorting Genetic Algorithm II (NSGA-II), and Monte Carlo simulation (MCS), collectively referred to as the DNN-GA-MCS strategy. The main objective is to ascertain complex process parameters while elucidating the intrinsic relationships between processing methods and material properties. To realize this, a numerical study on the PIM structural performance of an automotive front engine hood panel was conducted, considering fiber orientation tensor (FOT), warpage, and equivalent plastic strain (PEEQ). The mold temperature, melt temperature, packing pressure, packing time, injection time, cooling temperature, and cooling time were employed as design variables. Subsequently, multiple objective optimizations of the molding process parameters were employed by GA. The utilization of Z-score normalization metrics provided a robust framework for evaluating the comprehensive objective function. The numerical target response in PIM is extremely intricate, but the stability offered by the DNN-GA-MCS strategy ensures precision for accurate results. The enhancement effect of global and local multi-objectives on the molded polymer–metal hybrid (PMH) front hood panel was verified, and the numerical results showed that this strategy can quickly and accurately select the optimal process parameter settings. Compared with the training set mean value, the objectives were increased by 8.63%, 6.61%, and 9.75%, respectively. Compared to the full AA 5083 hood panel scenario, our design reduces weight by 16.67%, and achievements of 92.54%, 93.75%, and 106.85% were obtained in lateral, longitudinal, and torsional strain energy, respectively. In summary, our proposed methodology demonstrates considerable potential in improving the, highlighting its significant impact on the optimization of structural performance.

## 1. Introduction

Plastic injection molding (PIM) has been extensively utilized in the manufacturing process of carbon fiber-reinforced polymer (CFRP) products due to its excellent productivity, economic benefits, and high adaptability for various complex geometries [1,2]. In recent years, thermoplastic polymers have gradually occupied an important position in the application of high-performance structural materials due to their lightweight, excellent mechanical properties, and low cycle time in PIM processes [3,4]. Although CFRP materials offer alternatives to traditional metal materials, challenges still exist, pertaining to the anisotropic material properties inducing the stacking of long fiber composites, as well as the fiber orientation associated with the polymer flow during injection molding [5,6].

PIM constitutes an intricate, nonlinear, multiple-input, and multiple-output system. The input encompasses diverse factors such as mold configuration, machine characteristics, product geometrical features, process parameters, and polymer material characteristics, whereas the output primarily consists of manufacturing expenses, product quality, and molding efficiency [7,8]. The molds, machines, materials, and products are determined before processing, meaning the molding process parameters must be attentively set to increase productivity and prevent or decrease quality defects, computational consumption, and cost [9]. The process parameters in PIM have a decisive impact on these properties, but there is often a mutually restrictive relationship between them [10]. Therefore, it is a highly challenging task to accurately and quickly determine the molding process parameter settings to gain the quality requirements while ensuring that productivity and cost are acceptable.

Earlier, researchers used the Taguchi method and analysis of variance (ANOVA) to investigate the influences of warpage and shrinkage on product quality [11] (Oktem et al. [12], Tang et al. [13], and Kurt et al. [14]). Conclusions were drawn that shrinkage in thin-walled products is caused by fiber orientation distribution, which can be decreased by employing higher filling pressures and melt temperatures [15].

In the last two decades, advancements in computer technology have facilitated the integration of computer-aided engineering (CAE) simulation software (e.g., Moldex3D, Moldflow), which has been closely integrated into design optimization processes and thereby approved as an alternative scenario to obtain the optimal molding process parameters. These PIM numerical simulations often require the use of surrogate models or meta-modeling techniques to simplify complexity due to its computationally intensive nature. These technologies enable the identification of the optimal process parameter configuration within a limited number of simulations by constructing mathematical mapping while molding process parameters and optimizing targets.

Shi et al. integrated neural networks (NNs) and genetic algorithms (GAs) [16], where multi-layer neural networks were used to approximate the maximum shear stress and GAs were utilized to determine the optimal molding process parameters. Kurtaran et al. [17] and Kurtaran and Ezurumlu [18] also used NNs and quadratic polynomials to approximate warpage values and resolve the optimal molding process parameters. Ozcelik et al. [19] initially selected key process parameters using ANOVA and then used NNs and quadratic polynomials to approximate the warpage to resolve the optimal combination of molding process parameters. Zhang et al. [20] and Deng et al. [21] applied mode-pursuing sampling (MPS) algorithms for warpage reduction. Moreover, the multi-objective optimization based on Simulated Annealing Optimization (SAO) has been proven to be an effective approach for parameter optimization in PIM processes [22,23,24,25,26,27].

On this basis, methods combining approximation techniques such as radial basis function (RBF) networks [28], NN models [29,30], and Kriging models [31,32] with optimization techniques, for example, GA and sequential quadratic programming (SQP), have been widely employed in the literature for molding process parameters optimization. These innovative approaches strengthened multiple computational methods to increase the efficiency and precision of the optimization process. Their research underscored the versatility of combining machine learning techniques with traditional mathematical models to tackle complex engineering problems.

However, there remains the necessity for practical and in-depth conclusions beyond mere outcome parameters to facilitate the adjustment of process parameters in engineering under specific circumstances. Researchers such as Dimla et al. [33] Wang et al. [34] Kitayama et al. [35,36,37,38], and Guo et al. [39,40] have enriched similar domains by integrating cutting-edge advancements to enhance the quality and efficiency of products. The implementation of these sophisticated computational techniques within the background of PIM integrates a mix of machine learning networks, optimization technology, and computational physics. Although it is expected to substantially address the nonlinear, complex, and stochastic characteristics of CFRP, there remains a need for more substantive results and discussions.

Although there is potential for multi-objective optimization in the PIM process, as evidenced by Table 1, there remains an overall comprehensive disparity in understanding its inherent variability and integrability. Our goal is to elucidate the complex relationships between the processing method and material properties by employing advanced representation technologies, detailed analytical methodologies, and intelligent computational approaches. These complex relationships have been previously constructed through research; however, they are lacking in sufficient visualization in adjusting process parameters.

Our research primarily focuses on the identification of the performance of the numerical objective functions, for these functions play a significant role in affecting the mechanical properties of structures. These mechanical properties, conversely, affect subsequent innovative process controls, finally strengthening the overall structural performance. Our research aims to achieve innovative and significant advancements across critical domains, with relevant motivations summarized as follows:

- PIM mainly focuses on the multi-objective optimization of process parameters including the mold temperature, melt temperature, packing pressure, packing time, injection time, cooling temperature, and cooling time. Warpage is one of the major defects, and the optimal fiber orientation tensor (FOT) is considered to be maximized. Moreover, equivalent plastic strain (PEEQ) was considered to be optimized for structural performance. Z-score normalization metrics provided an advanced rigorous evaluation framework for the comprehensive evaluation of testing injection products.
- A deep neural network (DNN) was used to model complicated non-linear relationships between the product quality and various molding process parameters, which helps to quickly evaluate various parameter sets. Non-dominated Sorting Genetic Algorithm II (NSGA-II) enabled the simultaneous tuning of process parameters to achieve multiple objectives. Monte Carlo simulation (MCS) was utilized to simulate the various results probabilities under conditions of uncertainty, offering valuable insights into the robustness of selected process parameters.
- Subsequently, to attain high productivity, high product quality, and reduced computational expenditure, the DNN-GA-MCS focuses on multiple-objective optimization of process parameters in a PIM environment. This approach amalgamates various components, including sampling methodologies, computational simulations, metamodeling techniques, and sophisticated multi-objective optimization algorithms, with the aim of optimizing FOT, Warpage, and PEEQ.
- The Latin hypercube sampling (LHS) sampling strategy was employed to obtain a uniform and stratified set of training samples within the design space. With regard to these sampled points, numerical simulations are conducted using Moldflow to compute their respective responses. Subsequently, meta-modeling techniques are applied to construct response predictors on account of the sampled points and their corresponding responses. These predictors are designed to estimate the response of any training sample in a global design space. This multiple-objective design optimization approach was proposed to determine trade-off Pareto fronts.

In summary, the DNN-GA-MCS has been proposed, which includes sampling strategies, numerical simulations, metamodeling techniques, and multi-objective optimization algorithms. This integrated approach is specifically designed for the multiple-objective optimizations of the molding process parameters within PIM, aiming to enhance productivity, product quality, and cost-saving.

## 2. Finite Element Simulation Implementation Flow

**Figure 1:**
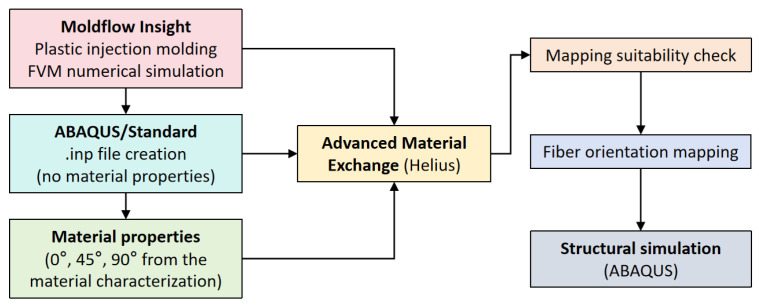
Numerical simulations implementation process of mapping operation flow in the Helius module for advanced material exchange.

To study the complex relationship between processing methods and material properties, the mechanical properties of materials were first studied. On the basis of the ASTM-D638 specification, the material properties of a polyamide-6 thermoplastic base polymer filled with 20% short carbon fiber (commonly referred to as PA6-20CF) were studied in laboratory experiments on the samples. To illustrate the effect of the local fiber orientation on the mechanical properties, the fiber orientation generated by the PIM was mapped into the CAE structural simulation element. Figure 1 reports the overall process, including FVM simulation, fiber orientation generation, material properties mapping, and FEM simulation stages. The program used consists of three steps, as described below.

In the first stage, we used the Auto desk Moldflow Insight (AMI) 2022 (San Rafael, CA, USA) program to simulate the thermoplastic injection molding (PIM) process and calculate the fiber orientation at the end of the filling/cooling step. Next, the fiber orientation was integrated into the orthogonal anisotropic material properties of PA6-20CF material, and these material properties were assigned to each element of the PIM simulation mesh according to its relevant fiber orientation tensor. Thirdly, advanced material exchange Helius 2019 (AME) was used to map the mechanical properties of elements onto the structural simulation mesh. In the final stage, the input file for ABAQUS 2017 (Dassault Systems, Velizy Villacopley, France) and the built mechanical property set based on meshed elements were established and run by the command windows.

### 2.1. Material Properties

**Figure 2:**
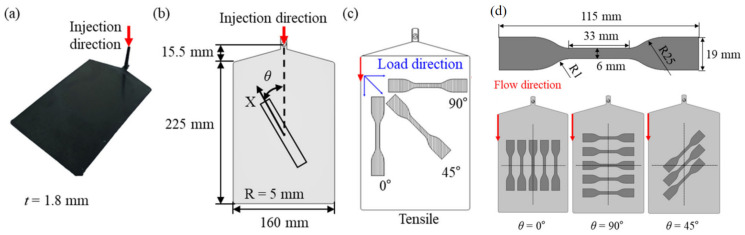
(**a**) PA6-20CF specimen samples manufactured by PIM, (**b**) schematic diagram of specimen samples with various fiber directions (*θ*) from the PIM samples (X as loading axis), (**c**) fiber arrangement and fiber orientation tensor (*θ*) in the sample of 0°, 45°, and 90°, (**d**) dimensions of ASTM-D 638-02a-IV type sample and slicing positions of tensile specimens from the plate. Reproduced from [5], MDPI, 2023.

The PA6-20CF material properties were derived from previous research by Lee et al. [5] and were inversely calibrated in FVM simulations to facilitate mapping into the FEM model. PA6-20CF materials reinforced with KN111 polyamide-6 (Kolon Plastics Inc., Gimcheon, Republic of Korea) [41] and Torayca T700S (Toray Carbon Fibers America Inc., Tacoma, WA, USA) [42] short carbon fibers by a weight fraction 20% were studied, and these material properties are shown in Table 2. The PA6-20CF plate specimen with a 1.8 mm thickness was manufactured by PIM of PA6-20CF pellets, and these specifications are shown in Figure 2. The whole procedure included material performance calibration, FVM simulation, fiber reorientation, material performance mapping, and FEM simulation stages. This method allows for uniform FOT to be achieved in the calibration area of the sample, thereby accurately estimating material properties related to direction.

**Figure 3:**
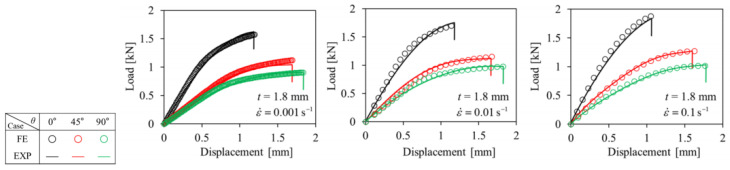
Numerical and experimental curves of PA6-20CF ASTM-D638 tensile specimens. Reproduced from [5], MDPI, 2023.

**Figure 4:**
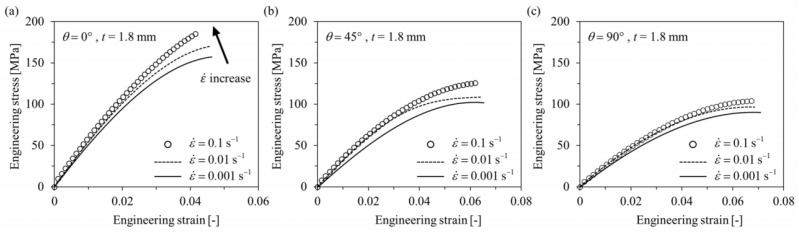
Tensile engineering stress–engineering strain curves of PA6-20CF ASTM-D638 tensile specimens of fiber orientation tensor (*θ*) as (**a**) 0°, (**b**) 45°, and (**c**) 90°. Reproduced from [5], MDPI, 2023.

Figure 3 reports the experimental and numerical results of tensile specimens of the PA6-20CF material in the ASTM-D638 standard. The accuracy and consistency of numerical simulation were verified. Figure 4 shows the engineering stress–strain curves of the PA6-20CF material under different orientations along 0°, 45°, and 90°. The material properties were inputted into the Moldflow Insight software as reported in Figure 3. Subsequently, by implementing the mapping calibration technique [6], as shown in Figure 4, the difference between the experimental load–displacement curve and the numerical simulation results was effectively reduced.

The material properties of the AA 5083 material have been obtained from previous research by Fonseca et al. [43]. Table 3 shows the mechanical properties provided by automotive parts manufacturing industrial partner, Donghee Industrial Co., Ltd. (Ulsan, Republic of Korea).

### 2.2. Thermoplastic Injection Molding Simulation Numerical Implementation

To describe the mechanical behavior of materials in different directions accurately, the Ramberg–Osgood flow stress model and the modified Hill’48 yield function were adopted, and material properties were interpolated, in which the *α* and *β* parameters correspond to the characteristics of the fiber direction (0°) and the transverse direction (90°), respectively. The yield function was scaled proportionally to account for the orthogonality of the injection-molded polymer. The mathematical expressions of the Ramberg–Osgood stress model and the modified Hill’48 yield function are shown as follows:

(1) $$\sigma =E^{1/n}{\left(K\right)}^{\left(n-1\right)/n}{\left(\varepsilon_{p,eff}\right)}^{1/n}$$

(2) $$\sigma_{eff}={\left[\frac{1}{2}\left\{{\left(\alpha \sigma_{11}-\beta \sigma_{22}\right)}^{2}+{\left(\beta \sigma_{22}-\beta \sigma_{33}\right)}^{2}+{\left(\beta \sigma_{33}-\alpha \sigma_{11}\right)}^{2}+6\left[{\left(\sigma_{12}\right)}^{2}+{\left(\sigma_{23}\right)}^{2}+{\left(\sigma_{31}\right)}^{2}\right]\right\}\right]}^{1/2}$$

where the *α* and *β* parameters are used to scale the Hill’48 isentropic yield function to consider different failure behaviors along the 0° direction (i.e., fiber direction) and 90° direction (i.e., matrix direction). Due to the orthogonality and anisotropy of the material model under consideration, directions 2 and 3 were assumed to yield consistent matrix-dominated mechanical and failure behavior. The fiber orientation was based on local elements and global mechanical properties calibrated on specimens, and the *α* and *β* parameters of each element of the mesh could be simulated. This fact means that each element of the mesh is considered an independent element with its own stress tensor. Based on the above discussion, we can conclude that each grid element is considered an independent entity with its unique set of mechanical properties and corresponding stress tensors. Within this framework, we further consider the potential impact of hydrostatic stress on material failure behavior. In the above equation, the specific role of the *α* and *β* parameters is that they reflect the significant improvement of the mechanical properties of the polymer matrix in lines parallel and perpendicular to the local flow direction due to the intervention of carbon fibers. The calculations are as follows:

(3) $$\begin{array}{cc} \alpha \left(\lambda_{I}\right)=\theta +\left[\frac{\alpha_{m}-\theta }{\lambda_{m,I}-\frac{1}{2}}\right]\left(\lambda_{I}-\frac{1}{2}\right) & \beta \left(\lambda_{I}\right)=\theta +\left[\frac{\beta_{m}-\theta }{\lambda_{m,I}-\frac{1}{2}}\right]\left(\lambda_{I}-\frac{1}{2}\right) \end{array}$$

Table 4 reports the material constants related to the above equation and refines the complete set of constants included in FVM simulations. The ARD-RSC model [5,6] was used to estimate the material properties of elements based on local fiber orientation estimation, as described below.

Recent studies have shown [5,6] that the Adaptive Rotation Diffusion Rotation Diffusion (ARD-RSC) model is highly accurate in predicting FOT during TIM processes. Compared with the traditional Folgar Tucker model [44], two improvements have been made. Specifically, the mathematical expression of the ARD-RSC model is as follows:

(4) $$\frac{\partial a_{ij}}{\partial t}=-\frac{1}{2}\left(w_{ik}a_{ki}-a_{ik}w_{kj}\right)+\frac{1}{2}\lambda \left({\dot{\gamma }}_{ik}a_{kj}+a_{ik}{\dot{\gamma }}_{kj}-{\dot{2\gamma }}_{kl}\left[a_{ijkl}+\left(1-k\right)\left(L_{ijkl}-M_{ijkl}a_{mnkl}\right)\right]\right)\;+\dot{\gamma }[2\left(c_{ij}-\left(1-k\right)c_{kl}M_{ijkl}\right)-2kc_{kk}a_{ij}-5\left(c_{ik}a_{kj}+a_{ik}c_{kj}\right)+10c_{kj}\left(a_{ijkl}+\left(1-k\right)\left(L_{ijkl}-M_{ijkl}a_{mnkl}\right)\right]$$

The former is associated with the interaction between fibers in polymer flow and thus is achieved through the fiber interaction function (*c_ij_*), which replaces the fiber interaction constant of the Folgar–Tucker model. In this context, *c_ij_* serves as a quadratic function, determined by both the fiber orientation tensor (*a_ij_*) and the deformation rate tensor (*γ_ij_*). The improved closure term determined by [*a_ijkl_* + (1 − *k*)(*L_ijkl_* − *M_ijkl_*⋅*a_mnkl_*)] plays a crucial role in fiber reorientation during the injection molding phase, where *L_ijkl_* and *M_ijkl_* are calculated as the products between the orientation tensor (*a_ij_*) of the eigenvalue and eigenvector components. The fiber reorientation constant k allows for scaling of the degree of fiber reorientation during the injection molding stage, ranging from completely free restricted reorientation (*k* ≈ 1) to high reorientation (*k* ≈ 0). Here, the reorientation constant was calibrated to *k* as 0.6 using the method applied by Quagliato et al. [45]. On this basis, FVM simulations were implemented in the Autodesk in sight 2022 environment. Table 5 provides the detailed configuration for numerical simulations in Moldflow.

### 2.3. Structural Analysis Simulation Model

**Figure 5:**
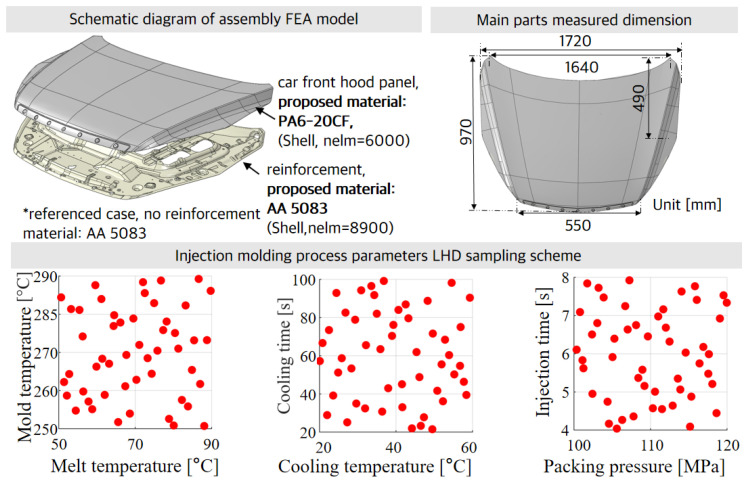
Schematic diagram of assembly FEA model with car front hood panel, main parts’ measured dimensions, and injection molding process parameter LHD sampling scheme.

The manufacturing process of a polymer–metal hybrid (PMH) car front hood panel commences with design and engineering simulations, wherein initial sketches and CAD models are developed and tested. Subsequent molding simulations direct the placement of material into a mold with reinforcement components to accurately form the PMH hood panel shape. Advanced Material Exchange Helius 2019 (AME) is employed to map the mechanical properties onto the structural simulation mesh, and the input file for ABAQUS 2017 is created and executed with specified boundary conditions. This process ensures proper alignment and functionality, culminating in rigorous quality inspections. This automotive front hood panel design concept was recently detailed by Bere et al. [46], where polymer materials and metal materials could be coupled to manufacture a front hood panel. Among them, Fonseca et al. [43] demonstrated the manufacturing method of a molded polymer–metal hybrid (PMH) cross member and verified its strength. Numerical results showed that the PMH model achieved an effective equilibrium between functional performance and PIM suitability. Figure 5 represents a schematic diagram of an assembly FEA model with a car front hood panel and the main parts’ measured dimensions.

In this section, PIM mainly focuses on multi-objective optimization of process parameters including the mold temperature, melt temperature, packing time, packing pressure, injection time, cooling time, and cooling temperature. The ranges for parameter settings as shown in Table 6. Warpage is one of the major defects, while the optimal fiber orientation tensor (FOT) is considered to be maximized; moreover, equivalent plastic strain (PEEQ) was considered as an aspect of structural performance to optimize. Considering the difficult-to-visualize 10-dimensional design space, we established the LHD sampling examples by picking out 35 samples in the 2D space, as shown in Figure 5.

The hood panel structural stiffness evaluation in the recent literature includes several load types: longitudinal bending, transversal bending, and torsion [47]. Evaluating transversal bending, longitudinal bending, and torsion on the hood panel is essential due to the diverse and dynamic forces encountered during vehicle operation. Transversal bending assesses the panel’s resilience to lateral impacts and side-to-side stresses, ensuring durability during cornering and side collisions. Longitudinal bending examines the panel’s capacity to withstand front-to-back forces experienced during acceleration, braking, and frontal impacts, thereby maintaining structural integrity under such conditions. The torsional analysis addresses the twisting forces that occur from uneven terrain and high-speed maneuvers, ensuring the hood panel’s alignment and functionality remain uncompromised. Incorporating these load evaluations is critical to ensuring the overall safety, performance, and durability of the hood panel in real-world driving scenarios.

**Figure 6:**
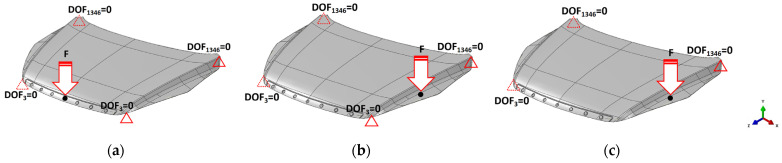
The boundary conditions used for evaluation are (**a**) lateral stiffness, (**b**) transversal stiffness, and (**c**) torsional stiffness.

For these loading conditions, the hood panel was assumed to be installed with its actual equipment including support points, rear hinges, and front bumper points. The standard for stiffness testing is the elastic deflection caused by the applied load. The overall testing mainly referred to the research of Bere et al. [46], and all contact surfaces used for the overall interface used tie constraint conditions. Support structural stiffness is defined as lateral stiffness, transverse stiffness, and torsional stiffness with degrees of freedom (DOF) ranging from 1 to 6, as shown in Figure 6, where 1, 2, and 3 represent translational constraints on the X-, Y-, and Z-axes, and 4, 5, and 6 represent rotational constraints on the X-, Y-, and Z-axes [48,49,50]. The Cartesian coordinate system was oriented with the X-axis as the longitudinal axis, the Y-axis as the lateral axis, and the Z-axis as the longitudinal axis of the hood panel. Therefore, to obtain the lateral and longitudinal stiffnesses, a vertical concentrate force of 600 N was applied, while for the torsional stiffness, the vertical force was approximately 100 N [46,50].

## 3. Design Optimization

### 3.1. Design Formula Explanation

In this study, we used multi-objective optimization methods to determine process variables in the injection molding process. Miettingen expounded on the measurement formula for multi-objective design optimization [51]. In this frame, *Obj* describes the objective function encompassing fiber orientation, warpage, and equivalent plastic strain. The design variables are denoted as *x_i_*, with upper and lower boundaries of *x_i_^U^* and *x_i_^L^*. The total number of design variables number is *n*, with *f*(*x*) representing the objective function to be optimized and constraints represented by *g_j_*(*x*), in conjunction with the number of constraints *n_con_*.

Minimize *obj_f_*, *obj_w_*, *obj_p_*,
S.T. *x_i_^L^* ≤ *x_i_* ≤ *x_i_^U^ i* = 1,2..., *n* *g_j_*(x) ≤ 0 *j* = 1,2..., *n_con_* (5)

### 3.2. Objective Function Formula

In this segment, multi-objective optimization was conducted through the evaluation of various objective functions, paying particular attention to the fiber orientation, warpage, and equivalent plastic strain. Firstly, simulation results values were collected from elements of the product part component. Afterward, local correlation variables and interrelated outcome responses were assessed by the Z-score normalization metrics and used as objective functions on the basis of the DNN-GA-MCS strategy. The standards established by these indices were utilized to assess the formability of product components and identify the corresponding structural performance. We then used these standards to determine the objective function, with the goal of optimizing the following response objectives.

Fiber orientation tensor: The fiber orientation generated during injection molding has a significant impact on the local material properties of the considered structure. Neglecting this impact can lead to a serious underestimation or overestimation of mechanical performance.

Warpage: Warpage represents the primary defect in PIM. This leads to the actual dimensions of the molded product deviating from the intended design specifications. To achieve high-quality products, efforts should be made to minimize warpage.

Equivalent plastic strain (PEEQ): PEEQ is a crucial parameter in the numerical simulation of failure analysis, particularly in the context of materials undergoing plastic deformation. It provides insights into the material’s deformation behavior and helps predict failure locations, making it an indispensable tool in engineering and materials science.

**Figure 7:**
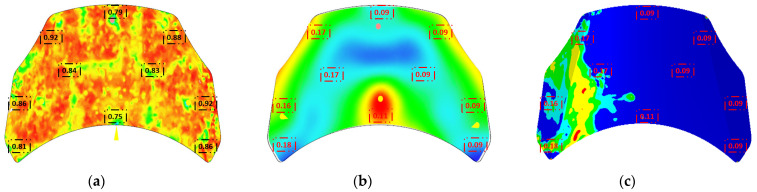
Definition of objective functions for evaluation in an example case of (**a**) fiber orientation tensor, (**b**) warpage, and (**c**) equivalent plastic strain (PEEQ).

To measure a composite value that combines multiple evaluation metrics including the maximum value, minimum value, the difference between the maximum and minimum values (range), and other relevant factors for determined regions, the standardized metric method using Z-score normalization was used with a weighted sum to combine these metrics. The calculation and standardization of the mean, standard deviation, Z-score normalization, and Z-score metric are as follows:

(6) $$\begin{array}{ccc} Obj_{f}=\{\frac{1}{n}\overset{nelm}{\underset{i=1}{\sum }}CI\left(\theta_{f}^{i}\right) & Obj_{w}=\{\frac{1}{n}\overset{nelm}{\underset{i=1}{\sum }}CI\left(\left|l_{w}^{i}\right|\right) & Obj_{p}=\{\frac{1}{n}\overset{nelm}{\underset{i=1}{\sum }}CI\left({\bar{\varepsilon }}^{i}\right) \end{array}$$

(7) $$CI\left(x\right)=\frac{x-\mu }{\sigma }$$

where *θ*, *l*, and *ε* represent the measured values of FOT, warpage, and PEEQ, *x* is the original data point, *μ* is the mean of the dataset, and *σ* is the standard deviation of the dataset. Moreover, the practical implementation is to collect the color of the pixel in the collection circle to determine the size of the value, and finally take the average value to determine the value of each circle. Figure 7 reports the definition of objective functions for evaluation in an example case of (a) fiber orientation tensor, (b) warpage, and (c) equivalent plastic strain (PEEQ).

## 4. DNN-GA-MCS Strategy

**Figure 8:**
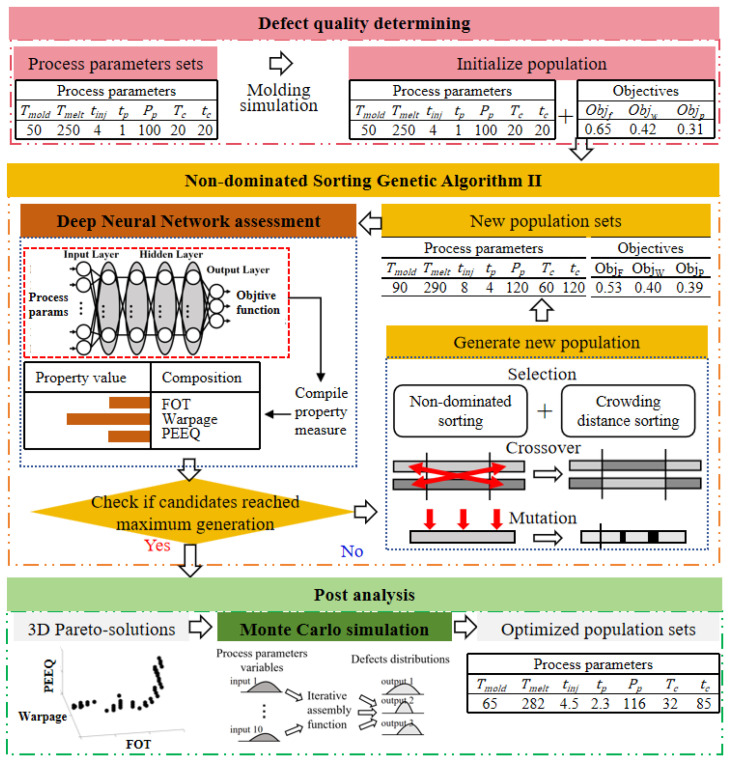
Schematic diagram of injection molding process parameter optimization process.

In this study, a surrogate modeling approach for multi-objective optimization in PIM is proposed, which employs a strategy using the integration of DNN-GA-MCS. This method synergistically combines machine learning and optimization techniques to enhance the potential for optimizing the fiber orientation tensor (FOT), warpage, and equivalent plastic strain (PEEQ). An integrated schematic diagram of the DNN-GA-MCS strategy working process is presented in Figure 8. The surrogate model offers an effective approximation to the intricate systems, applying expedited optimization and global sensitivity analysis simultaneously decreasing computational expenses. A DNN was trained to approximate the structural performance probabilities associated with various process parameters. Subsequently, the approximated objectives were utilized as inputs for Non-dominated Sorting Genetic Algorithm II (NSGA-II) to obtain optimized process designs based on the Pareto optimal set. Thereafter, Monte Carlo simulations (MCSs) were conducted to analyze the optimal process parameters, assessing their frequency characteristics under uncertain conditions. This comprehensive approach facilitates the identification of robust and efficient process parameter configurations, contributing to the advancement of PIM technology. This approach involved a detailed analysis of the relationships between input variables (process parameters) and desired outcomes (response target), allowing for the precise determination of key process parameters that significantly influence each response target. By leveraging the predictive capabilities of the DNN, we could model these complex interactions and generate accurate predictions of performance metrics. The Genetic Algorithm then iteratively refined these parameters, balancing multiple objectives to achieve an optimal configuration. This systematic methodology ensured that each process parameter was finetuned to enhance the overall performance, leading to improved efficiency and effectiveness in the splitting of process parameters.

Finally, the optimized process parameters were additionally verified using the FEA method to understand and predict the material’s response under various PIM process parameters. The results of numerical simulation highlight the practicality of the DNN-GA-MCS method in addressing the challenges of PIM engineering applications, especially in providing the robustness and reliability of process parameters. The key technology of this study lies in the PIM structural performance analysis at the numerical processing level, which plays a crucial role in determining the CFRP mechanical properties.

### 4.1. Deep Neural Network (DNN)

The proposed DNN model effectively converts complex process parameters into interpretable mathematical models by leveraging its multi-layer architecture, nonlinear activation functions, and extensive training on diverse datasets. The DNN model begins with multiple layers that extract high-level features from the raw input data, which consists of complex process parameters. This study uses deep neural networks (DNNs) as meta-models to approximate the RSM models, particularly for key samples in the design space [52,53]. During training, the DNN learns the optimal weights and biases that minimize the error between the predicted outputs and the actual target values. The use of nonlinear activation functions (ReLU) in the neurons allows the DNN to model the intricate non-linear interactions among the molding process variables. The depth of the network, with multiple layers of neurons, allows the DNN to build hierarchical representations of the input data. Each successive layer captures increasingly complex interactions and dependencies between the process variables. The network’s architecture inherently captures interaction terms between variables through the combination of weights and activations in different layers. It addresses nonlinear interaction effects by capturing intricate dependencies between process variables through its hierarchical and flexible structure. This capability enables the DNN to provide accurate and interpretable predictions, making it a powerful tool for understanding and optimizing complex processes.

**Figure 9:**
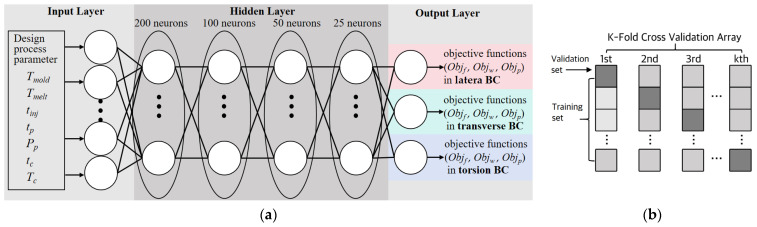
(**a**) Schematic diagram of injection molding process parameter DNN structure modeling and (**b**) K-fold cross-validation array. We used K-fold cross-validation to ensure database consistency before training and prevent overfitting. In the K-fold cross-validation process, the dataset is divided into k groups and then trained or validated according to predetermined distribution standards.

The specific layout of the DNN structure used in this study is shown in detail in Figure 9a. The DNN model has an input layer with seven neurons, which represent process parameters including the mold temperature, melt temperature, packing pressure, packing time, injection time, cooling temperature, and cooling time. The output layer has three neurons for the objective functions of fiber FOT, warping, and PEEQ. Referring to previous research, the performance of the model could be enhanced by applying a DNN structure with five hidden layers to achieve deeper and wider networks [54,55,56]. To improve the fitting ability and generalization performance of the model, 128, 256, 512, 256, and 128 neurons in each hidden layer were used to construct a hierarchical feature extraction mechanism. We used the rectangular linear unit (ReLU) activation function as a non-linear mapping tool for the hidden layer, which helped to reveal the inherent non-linear patterns in the data and enhance the expressive power of the feature space [57,58]. The introduction of the ReLU function enables the network to effectively process and transmit complex feature information while maintaining computational efficiency. This setting could accurately capture the complex interactions between the input and output variables [59,60]. In the actual operation process, the TensorFlow deep learning platform and the Keras framework were used to build and train the above network structure. In summary, the DNN architecture proposed in this study demonstrates good adaptability and superior performance in nonlinear regression tasks, laying a solid technical foundation for further exploration in related fields.

**Figure 10:**
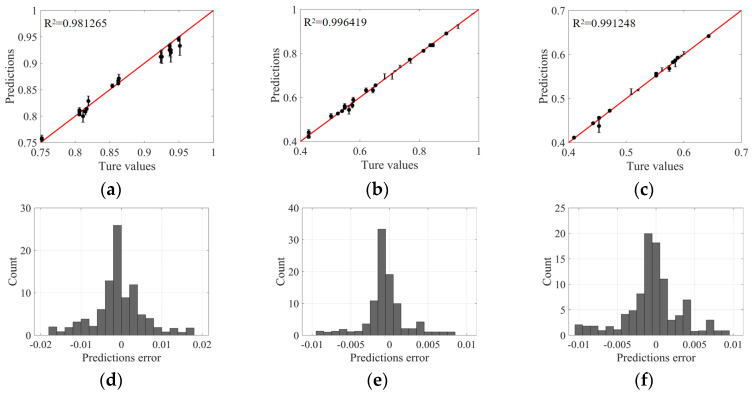
The DNN validation results revealed a close correlation between predicted values and true values, as well as the relationship between approximation error and R-squared. Sub-figures show the training dataset (**a**–**c**) for FOT, warpage, and PEEQ and the test dataset (**d**–**f**) for FOT, warpage, and PEEQ. In these sub-figures, the circles represent the predicted mean of the DNN model, the error bars represent the standard deviation proving the reliability of the DNN model predictions, and the bar distribution represents the identification of collected data falling inside the range, demonstrating the setting reliability of the DNN model.

To improve the generalization ability of DNN models, a K-fold cross-validation array was adopted to partition the dataset. As shown in Figure 9b, we allocated 80% of the total dataset to the training sets and the remaining 20% to the test sets. These divisions ensured that the DNN model could access rich and representative samples during the training phase. The Adam optimizer with a set of appropriate hyperparameters with a learning rate of 0.001, execution of 1000 periods, and batch size of 1000 was executed. The adjustment of these parameters aims to accelerate the convergence speed of the model and enhance its predictive performance on unknown data. As shown in Figure 10, the fully trained DNN model exhibits excellent prediction accuracy. Specifically, the model achieved a global average absolute error (MAE) of 2.32 × 10^−5^ and a mean square error (MSE) of 5.1 × 10^−3^. These quantitative indicators clearly reflect the high consistency between the predicted values of the model and the actual parameter values, proving the effectiveness and reliability of our proposed DNN model in prediction tasks. In summary, this study successfully constructed a DNN model with strong generalization ability through carefully designed data-partitioning schemes and optimization strategies. Its performance in nonlinear regression tasks is satisfactory, providing strong technical support for further research and application in related fields.

Two metrics, the mean square error (MSE) and the mean absolute error (MAE), were used to quantitatively assess the predictive accuracy of DNN models. The MAE provides an intuitive understanding of the average magnitude of residuals, while the MSE provides an overall evaluation of residual variance. The combination of the two metrics can help to analyze the performance of DNN models in prediction tasks more comprehensively, thereby guiding further optimization and adjustment of the model. The mean absolute error (MAE) is a direct indicator of the deviation between predicted values and true values. It reflects the overall degree of deviation in model predictions by calculating the average of all absolute values of prediction errors. The mean squared error (MSE) is a commonly used error measure that evaluates the performance of a model by calculating the average squared prediction error. MSE not only focuses on the size of errors but also emphasizes the impact of larger errors, as the square operation amplifies these errors. This calculation formula is as follows:

(8) $$MSE=\frac{1}{n}\overset{n}{\underset{i=1}{\sum }}{\left(y_{i}-\hat{y_{i}}\right)}^{2}$$

(9) $$MAE=\frac{1}{n}\overset{n}{\underset{i=1}{\sum }}\left|y_{i}-\hat{y_{i}}\right|$$

where *Ŷ_i_* signifies the predicted value of *Y*, *Y_i_* represents the real value of *Y*, and *n* denotes the overall number of samples.

### 4.2. Non-Dominated Sorting Genetic Algorithm II (NSGA-II)

**Figure 11:**
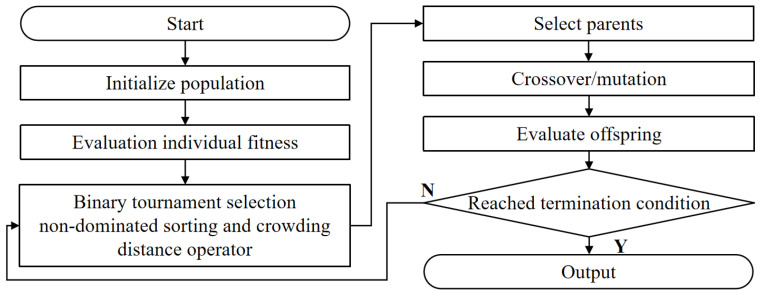
Schematic diagram of NSGA-II workflow.

In this section, we explain the methodology for employing NSGA-II to navigate the multi-dimensional search space, addressing the trade-offs between the three structural responses. We also outline the criteria and methods used to evaluate the quality and feasibility of the obtained Pareto optimal solutions, ensuring a comprehensive understanding of the optimization process. As shown in Figure 11, NSGA-II introduces a survival selection process within the overall framework of the genetic algorithm to meet the special requirements of optimization problems. This process involved formulating these responses as objective functions and defining relevant design variables. An initial random population of solutions was generated, each evaluated based on the objective functions using engineering models. Solutions were sorted into non-dominated fronts based on Pareto dominance and assigned crowding distances to maintain diversity. Selection for reproduction favored solutions from lower fronts and those with higher crowding distances. Genetic operations, including crossover and mutation, produced offspring, forming a new combined population, again sorted by non-dominated sorting and crowding distance. This iterative process continued until a stopping criterion was met, resulting in a final population of Pareto optimal solutions. These solutions, forming a Pareto front in the three-dimensional objective space, provided a comprehensive understanding of trade-offs among structural responses, aiding in selecting the most appropriate solutions.

The core of NSGA-II lies in identifying non-inferior solutions in the Pareto optimal set, which involves balancing multiple conflicting objective functions. These objective functions include minimizing fiber orientation tension (FOT), warping, and equivalent plastic strain (PEEQ). The algorithm uses the crowding distance as a measure to evaluate the diversity of the solution space, ensuring that the diversity of the population can be maintained during the selection process. The entire optimization process went through 2000 iterations to ensure that the algorithm had sufficient opportunities to explore the solution space and find satisfactory non-inferior solutions. The implementation of NSGA-II was executed using the TensorFlow library via the Keras frame. In summary, NSGA-II demonstrated its strong ability to deal with complex multi-objective optimization problems in this study, providing strong technical support for the optimization of process parameters. By combining it with deep learning platforms, we not only improve the operational efficiency of the algorithm but also broaden its application prospects in the field of engineering optimization.

### 4.3. Monte Carlo Simulation (MCS)

**Figure 12:**
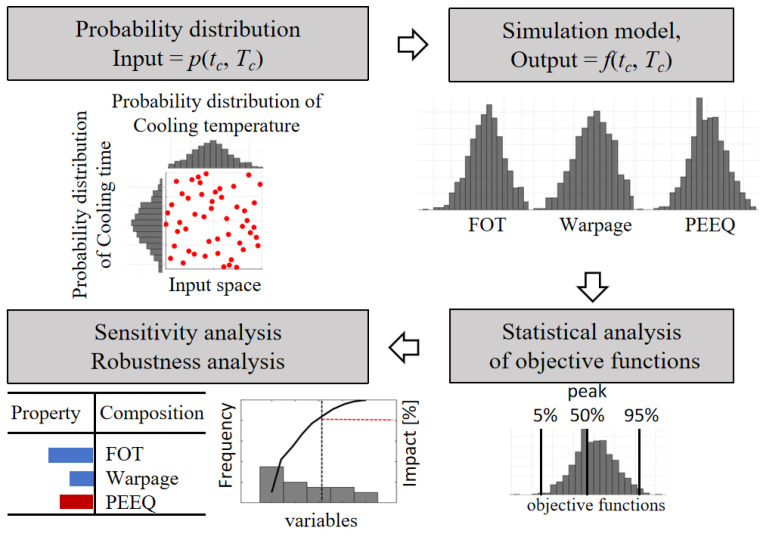
Workflow of Monte Carlo simulation.

This article provides a detailed explanation of the implementation process of MCS. Firstly, a series of key process parameter variables were established and an MCS model was constructed to map the intrinsic characteristics of the input space system. Subsequently, based on the preset probability distribution functions of each variable, a random input dataset was systematically generated. These data were then injected into the model to produce a series of frequency distribution results. Through an in-depth analysis of these results, uncertainty factors that may affect material performance characteristics can be evaluated. Ultimately, by comprehensively considering these results, the probabilities of various possibilities can be quantified and provide a scientific basis for subsequent decision-making processes. Figure 12 shows the workflow of MCS.

## 5. Numerical Results and Discussions

This section details the results of implementing the suggested approach in the PIM process. Initially, the DNN model was verified and we constructed an approximate surface that could illustrate the complex correlation between the object response and process parameters. Subsequently, NSGA-II was applied to the exploration of Pareto optimal solution sets, confirming the effectiveness of the multiple structural performance optimization. In addition, the MCS further evaluated the uncertainty level of the obtained solution and highlighted the improvement in process stability and reliability. This series of research findings not only validates the effectiveness of the proposed method but also provides a solid theoretical basis and practical guidance for the optimization of PIM processes.

### 5.1. Approximation of RSM Results by Deep Neural Network (DNN) Modeling

**Figure 13:**
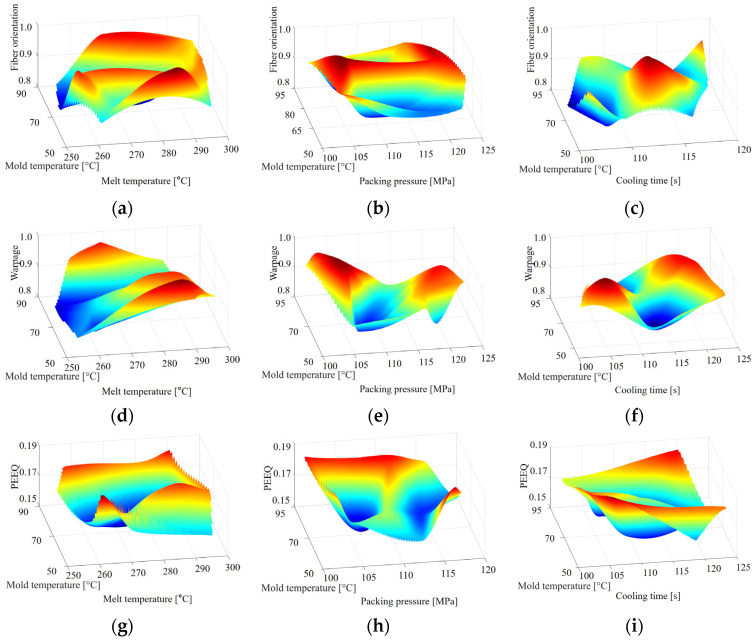
Approximate RSM model for the melt temperature, mold temperature, packing pressure, and cooling time variables with structural performances, depicting the response relationship across FOT, warpage, and PEEQ. (**a**)–(**i**) Distinct variables for each structural performance. Each sub-figure shows a unique relationship, describing the diversifications within DNN models under deliberation.

This study used the Latin hypercube design (LHD) strategy to generate 264 sets of ABAQUS simulation process parameters applied to experimental run sampling points. With these planned design combinations, a training dataset was constructed that revealed nonlinear interaction effects between defined process variables. Due to the large quantity of data in the 7-dimensional design space, we established a response surface methodology (RSM) diagram for each process parameter in the 3-dimensional design space. Figure 13 shows the response relationship between the approximate model as a process parameter and the three objective functions, indicating that by combining the inherent mechanisms of LHD design and machine learning algorithms, the proposed DNN model can effectively transform intricate molding process parameters into explainable mathematical models. Therefore, exploring the optimal solution of the splitting molding process parameters coupled with the RSM and solving the industrial problem of searching for the optimal molding process parameters has become the core goal of constructing the mathematical model.

Within the framework of applying RSM to process parameters, as shown in Figure 13, we conducted a detailed examination of three objectives. Each individual response objective was thoroughly analyzed through its own process parameters, revealing unique process parameters associated with each response target. These figures are divided into several sub-figures, each specifically representing a different type of response objective. We took the mold temperature, melt temperature, packing pressure, and cooling time as examples of design variables, and used the approximate Response Surface Methodology (RSM) model to depict the response relationship between FOT, warpage, and PEEQ. By analyzing Figure 13a,d,g, we observed that the mold temperature and melt temperature have specific effects on these three structural properties. In the mold temperature range of 60 to 85 °C, its impact is much smaller compared to the melt temperature. For the melt temperature, the optimal range could be identified between 260 and 270 °C. Similarly, the optimal packing pressure was likely between 105 and 110 MPa, and for cooling time, the optimal range was 110 to 115 s. However, the mold temperature demonstrated complexity with numerous local optima across all sub-figures, necessitating precise global optimization through the proposed method to effectively handle these complex variables.

### 5.2. Process Parameters Optimization Results by Nondominated Sorting Genetic Algorithm-II (NSGA-II)

**Figure 14:**
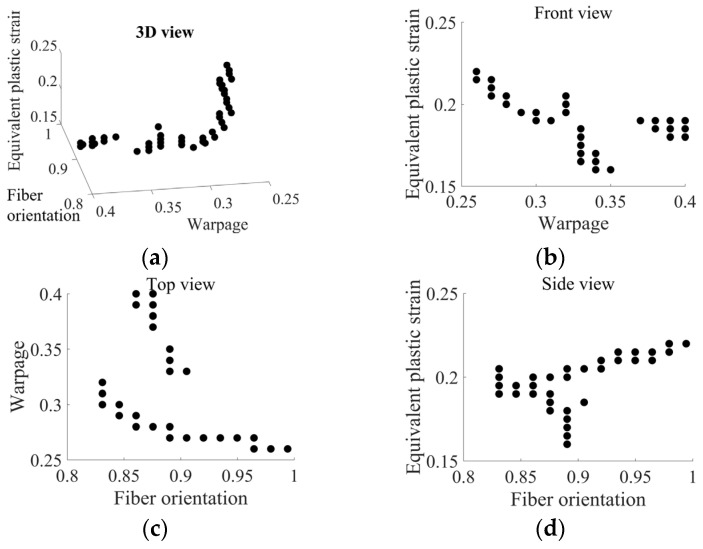
The Pareto optimal set contains multiple optimal responses with (**a**) 3D view design space section, (**b**) front-view section, (**c**) top-view section, and (**d**) side-view section.

This study uses NSGA-II to determine the optimal molding process parameters, aiming to simultaneously optimize three different types of structural responses. Figure 14 shows the outcomes gained by NSGA-II, in which each space point represents a Pareto optimal solution set containing multiple optimal responses. Since this study innovatively considered three objectives, including FOT, warping, and PEEQ, the Pareto optimal set manifests as a more complex three-dimensional space. Comparing the multi-objective optimization results with the mean value of the training set, all objectives are more ideal within the Pareto optimal set range. These outcomes indicate that the proposed method employing the DNN-GA-MCS strategy efficiently identifies optimal design variables by considering both global and local optima, while simultaneously optimizing the likelihood of achieving three structural responses.

We applied statistical tests to determine the significance of the observed improvement post-optimization. By comparing the objective function considering the optimization results with the average data and some special single excellence cases in the training set, we present Table 7 to display the process parameter optimization results. Compared with the training set mean value, the objectives were increased by 8.63%, 6.61%, and 9.75%, respectively. The verification of prediction accuracy for objectives at the optimized solution is summarized in Table 7, which indicates that the relative absolute errors for all objectives at the optimized solution are below 2.3%. This confirms and verifies the prediction accuracy.

### 5.3. Pareto Chart Results by Monte Carlo Simulation (MCS)

**Figure 15:**
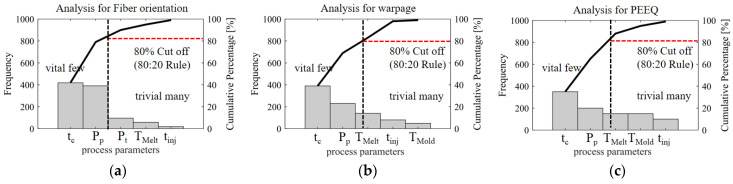
Pareto chart of types of the frequencies for various molding process parameters of multi-objectives of (**a**) fiber orientation tensor (FOT), (**b**) warpage, and (**c**) equivalent plastic strain (PEEQ).

The Pareto chart results shown in Figure 15a–c provide a detailed explanation of the frequency distribution of various molding process parameters in multi-objective optimization. By conducting an in-depth analysis of the frequency of these parameters, the study aims to determine the cumulative total frequency of multi-objective problems and their corresponding occurrence rates. The raw data are first organized into statistical tables and then rearranged in descending order and presented in the Pareto chart template charts. These charts combine a bar chart and a line: where the bar chart displays the independent frequencies of each parameter in descending order, while the line chart depicts the trend of the cumulative total number of samples. A special cutoff line of 80% is also marked in the figure to verify the submission of the 80/20 rules and identify the key minority factors that need priority observation located below the critical line.

Among them, the process parameters that have an important influence on FOT, warpage, and PEEQ are melt temperature, injection time, cooling temperature, cooling time, packing pressure, and packing time. Specifically, to control the fiber orientation tensor more accurately, more precise manufacturing tolerances should be used by the process parameters such as packing time and packing pressure. On the other hand, in order to control warping more accurately, process parameters such as packing pressure and packing time should adopt more precise manufacturing tolerances. Similarly, to control the PEEQ more accurately, more precise manufacturing tolerances should be considered for packing pressure and packing time. This outcome offers valuable guidance for engineers to accurately establish tolerance levels.

Previous researchers used a simpler Taguchi method to achieve similar results [12,61,62]. Chen and Kurniawan investigated a two-phase optimization study utilizing the Taguchi Method and PSO-GA [63]. Specifically, in the first phase, the Taguchi Method was utilized to determine the initial process parameters. In the second phase, the PSO-GA was utilized to obtain the optimal process parameters. However, it should be noted that despite the effectiveness of the Taguchi Method in finding the best parameter combinations, it does not necessarily find the absolute optimal values for the individual optimal process parameters.

### 5.4. Numerical Optimization Results

**Figure 16:**
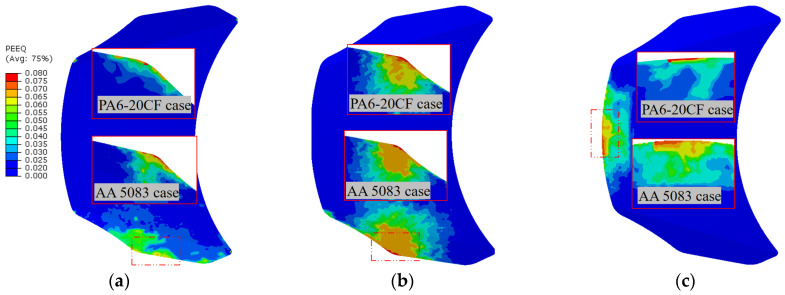
Comparison of PA6-20CF PMH and AA 5083 material case numerical optimization results of equivalent plastic strain (PEEQ) distribution with three boundary conditions of (**a**) lateral, (**b**) transverse, and (**c**) torsion.

A comparative analysis of stiffness was conducted for the AA 5083 hood and the PA6-20CF PMH hood with similar designs, and the analysis results clearly indicate that the PA6-20CF PMH hood has better stiffness performance than the AA 5083 hood with the same specifications. Figure 16 shows the after-mapping local stiffness-related results of the optimal process parameter scenario for (a) lateral stiffness, (b) transversal stiffness, and (c) torsional stiffness magnitude values, mainly encompassing the PA6-20CF PMH and AA 5083 numerical optimization results of equivalent plastic strain (PEEQ) distribution with three boundary conditions. The mass of the CFRP front hood panel is reduced by 0.72 kg, which is a reduction of 16.67% compared to the AA 5083 scenario. Meanwhile, considering the boundary conditions of (a) lateral, (b) transverse, and (c) torsion, achievements of 92.54%, 93.75%, and 106.85% were realized in the objective functions based on the Z-score normalization metric. The findings indicate that this investigated method enables users to accurately and swiftly select the optimal molding process parameter configurations. Moreover, compared with the training set average values, fiber orientation tensor (FOT) objective function values increased by 12.47%. Meanwhile, the values of the warpage and equivalent plastic strain (PEEQ) objective functions decreased by 10.75% and 7.34%, respectively, demonstrating significant enhancements in addressing structural performance optimization variations.

**Figure 17:**
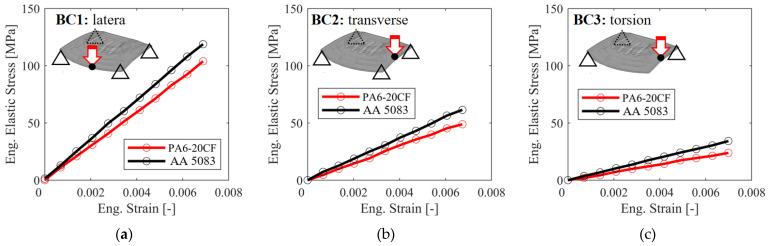
After-mapping local stiffness results of the multi-boundary conditions for (**a**) lateral, (**b**) transverse, and (**c**) torsion.

Figure 17 shows the after-mapping local stiffness-related results of the feature shapes for (a) lateral stiffness, (b) transversal stiffness, and (c) torsional stiffness magnitude values. According to the results shown in Figure 17, the PA6-20CF PMH hood has a higher stiffness than the AA 5083 hood and has the highest transverse and lateral stiffness. In terms of torsional stiffness, comparable results are obtained. The transverse stiffness of the composite hood is improved compared to the AA 5083 hood: the average stiffness is less affected by the material change and decreases by 16.2%. Due to the proximity and location of the load application points, the strain value results of the PA6-20CF PMH case showed significant values in the transverse load conditions compared to the transverse and torsion load conditions. The transverse stiffness of the PA6-20CF PMH hood is higher than both the transverse and torsional stiffness.

**Figure 18:**
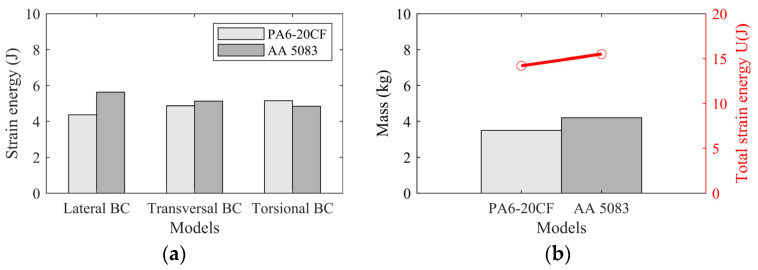
Numerical optimization results of PA6-20CF PMH case and AA 5083 case of (**a**) comparison of strain energy values under lateral, transverse, and torsional conditions and (**b**) comparison of total strain energy value and weight value.

FEA strain energy analysis was performed on each model, and the results are presented in Figure 18. In this process, we introduced the concept of total strain energy as a key indicator for regulating the overall stiffness of the model. By quantifying the energy absorbed and stored in a material as it deforms under an applied load, the potential stored energy of the structure can be determined by analyzing the strain energy within the model, allowing targeted modifications to be made to enhance stiffness. Specifically, in the comparative study, we found that compared to the traditional AA 5083 front hood panel case, the newly designed solution successfully achieved improvements in lateral, longitudinal, and torsional performance by 92.54%, 93.75%, and 106.85% while maintaining structural integrity. This achievement is visually shown in Figure 18a. Furthermore, Figure 18b reveals another important finding: under the same weight-reduction effect, our design also significantly improved the total strain energy, achieving a growth of 12.32%. The implementation of this achievement is attributed to the use of the topology optimization module in ABAQUS 2017 software. As demonstrated in previous studies [43], the total strain energy can be evaluated through multiple iterations to achieve an accurate assessment of a material’s response under load. In this study, however, we utilized only the first iteration step for the evaluation. This approach simplifies the analysis while still providing a sufficiently accurate estimate of the total strain energy, facilitating the examination of initial deformation characteristics.

The observed improvements in stiffness performance of the PA6-20CF plastic structure, when optimized by process parameters compared to the aluminum structure, are due to several key factors. Firstly, the inclusion of 20% short carbon fiber significantly enhances the polyamide-6 thermoplastic base polymer material’s elastic modulus, thereby increasing its resistance to deformation under load. Secondly, the precise control of process parameters, including cooling rates and fiber orientation, also plays a crucial role in achieving a more uniform and robust structure. The optimization of process parameters ensures that forces are efficiently distributed across the structure, minimizing PEEQ concentrations and improving overall rigidity. Furthermore, the inherent properties of polyamide 6, such as higher tensile strength and improved fatigue resistance, contribute to its superior performance under dynamic loading conditions. These combined factors result in a thermoplastic base polymer structure with enhanced stiffness and durability, surpassing the performance of traditional aluminum counterparts.

## 6. Discussion

Carbon fiber-reinforced polymer (CFRP) components with precise geometric shapes are manufactured through plastic injection molding (PIM), yet challenges exist in adapting to design changes. The developed DNN-GA-MCS strategy combined triple structural performance occurrence metrics data gathered from the PIM process to contrast structural performance characteristics for various process parameter combinations and construct a response surface model (RSM). This method cleverly integrates machine learning networks with global search algorithms, proposing a natural mechanism for optimization and explainable transition.

Our study explored the fields of PIM processes, material properties, and numerical simulations. By constructing a deep neural network (DNN), the response surfaces of the high-degree non-linear coupling relationship between molding process parameter combination variables were meticulously approximated, enabling incremental learning. Non-Dominant Sorting Genetic Algorithm II (NSGA-II) adopts a sequential sampling strategy to achieve dual objectives of local and global optimization objectives. Monte Carlo simulation (MCS) analysis based on the Pareto optimal set enhanced the stability phase of the PIM process. Design constraints defined as structural performance were rigorously measured and assessed employing the Z-score normalization metric evaluation framework. By identifying discontinuous Pareto boundaries, the optimal combination of process parameters was obtained to achieve the optimal structural performance. We discussed contributions in the following fields:

- We explored precision processing methods, exemplified by plastic injection molding (PIM), with triple structural performance optimization under various boundary conditions. Through the advancement of analysis, characterization, and modeling techniques, this study has deeply recognized and predicted the material behavior in the molding process parameter of PIM, revealing its unique characteristics and potential applications.
- The optimal combination of process parameters was determined through a surrogate model method that integrates the deep neural network, genetic algorithm, and Monte Carlo simulation (DNN-GA-MCS) strategy.
- The DNN model was adopted to establish the complex and non-linear characterizations of the PIM process. This model was designed to approximate the practical relationship between triple structural performance and the intricate process parameter combinations, therefore constructing the approximate RSM surface for subsequent research.
- The design constraint, considering the multiple structural performances of the model under various boundary conditions, was quantitatively evaluated employing the Z-score normalization metric framework to assess absolutely complex composite objective functions to ensure strict assessment.
- Considering both global and local optima is crucial for a comprehensive and effective optimization process, ensuring high-quality solutions and avoiding premature convergence. The proposed DNN-GA strategy leverages the strengths of both DNN and GA to enhance the optimization of structural responses. The DNN provides accurate and efficient evaluations, while the GA ensures robust exploration and exploitation of the search space. This synergistic approach results in a dynamic, adaptive, and multi-objective optimization process that effectively identifies optimal variables and structural responses.

The findings underscore the actual significance of the approach and validate the effectiveness of the DNN-GA-MCS-based surrogate modeling methodology in delivering reliable and robust molding process parameter selection for complex PIM manufacturing applications. Yet, a few restrictions exist:

- Initially, explaining the results of anisotropic FEA model analysis might be challenging. Although FEA models constitute a valuable tool for analyzing anisotropic material characterization, they necessitate meticulous focus on the precise definition and input of material properties. In this study, the fiber reorientation constant used k = 0.6 within the range of the injection stage, where a more precise definition needs to be studied.
- On the other hand, advanced characterization employs anisotropic mapping procedures, which precisely capture the anisotropic behavior of alloys using the Ramberg–Osgood model and the anisotropic Hill’48 yield function. Several issues, including material coefficient accuracy, numerical stability, convergence, and mesh sensitivity, have been addressed. However, the study did not cover boundary condition sensitivity, microstructural effects, and certain environmental factors with minimal influence.
- Additionally, DNN, GA, and MCS are computationally intensive, necessitating significant resources. Integrating these methods can lead to increased computational costs in terms of both resources and time. The investigative nature of GA further complicates risks related to generalization and robustness.
- At last, implementing this hybrid approach integrating DNN, GA, and MCS is intricate and necessitates a high level of expertise in each field. This challenge is particularly obvious when dealing with high-dimensional data and complex suitability landscapes.

To ensure the reliability and validity of our optimization results, we adopted a multi-faceted approach. This included comparing the optimization results with the aluminum-referenced model to validate performance improvements; applying statistical tests to determine the significance of observed improvements and using spider plots to compare optimization results with average and minimized data from the training set; verifying results through simulations and real-world tests where feasible to confirm practical applicability; and using metrics such as approximation error, R-squared, MAE, and MSE to quantitatively evaluate the DNN models’ prediction accuracy. Additionally, we performed cross-validation using the approximate RSM model, with each subplot depicting different relationships, to assess the generality and robustness of the optimized parameters

The proposed DNN-GA method effectively translates into practical benefits for manufacturers by enabling the selection of optimal process parameter configurations with high precision and efficiency. By leveraging the predictive power of deep neural networks to model complex relationships between molding process parameters and desired results, combined with the optimization capabilities of genetic algorithms, this method allows for the systematic exploration and identification of parameter settings that maximize performance indicators, including the increase in the fiber orientation tensor value and the maximization of quality related to equivalent plasticity, as well as the cost benefits related to subsequent processing associated with the reduction in warpage. This integration not only accelerates the decision-making process but also enhances the robustness and adaptability of manufacturing processes, ultimately leading to improved product quality, reduced operational costs, and increased competitiveness in marketing.

## 7. Conclusions

This study presents an innovative methodology aimed at addressing and optimizing multiple structural performance metrics. It incorporates the use of a deep neural network (DNN) for advanced response surface model (RSM) characterization, Non-dominated Sorting Genetic Algorithm II (NSGA-II) integrated with an intricate model for multi-objective optimization, and Monte Carlo simulations (MCSs) to analyze the robustness of the outcomes. This approach enables the determination of intricate molding process parameters and elucidates the complex relationship between processing methods and structural performances.

In the case study of this article, an equivalent polymer–metal hybrid (PMH) engine hood was compared with a PA6-20CF carbon fiber-reinforced polycarbonate fiber (CFRP) hood with similar geometry as the AA 5083 material design. The mechanical behavior of the PA6-20CF PMH hoods was analyzed in terms of static stiffness and strain energy monitoring in three different points for lateral, longitudinal, and torsional loads. Numerical analysis and high-precision validation of RSM technology indicated that the combination of plastic injection molding (PIM) process parameter variables and the intelligent optimization technique is an ideal process choice for producing high-quality PA6-20CF PMH parts. By replicating the design of suitable composite material products, the designed PA6-20CF PMH hood achieved excellent performance while maintaining similar or higher stiffness. The composite engine hood manufactured based on PIM is 16.67% lighter than the AA 5083 hood and showed a decrease in lateral stiffness by about 18.2%. In addition, achievements of 92.54%, 93.75%, and 106.85% were achieved in lateral, longitudinal, and torsional strain energies, respectively.

To summarize, this research underscores the effectivity of the integrated DNN-GA-MCS strategy in tackling the challenges inherent in the PIM process, with the complex molding process parameters associated with injection molding demonstrating particular promise within this integrated approach.

## Figures and Tables

**Table 1:** Representative literature of prediction of PIM optimal process parameters.

Literature	Method	Input	Output
Shi 2003 et al. [16]	BPN, GA,	*T_mold_*, *T_melt_*, *t_inj_*, *P_i_*	shear stress
Zhao 2015 et al. [26]	Kriging	*T_melt_*, *t_inj_*, *t_p_*, *p_p_*, *T_c_*, *t_c_*	shrinkage, sink marks
Kurtaran et al., 2005 [17]	BPN, QP	*T_mold_*, *T_melt_*, *t_p_*, *P_p_*, *t_c_*	warpage
Ozcelik et al., 2006 [11]	ANOVA, NN, QP	*T_mold_*, *T_melt_*, *t_p_*, *P_p_*, *t_c_*, *t_r_*, *l_g_*	warpage
Zhang et al., 2009 [20]	MPS	*T_mold_*, *T_melt_*, *t_inj_*,	warpage
Gao and Wang, 2009 [32]	Kriging	*T_mold_*, *T_melt_*, *p_f_*	warpage
Shi et al., 2010 [22]	BPN	*T_mold_*, *T_melt_*, *t_inj_*, *t_p_*, *P_p_*, *t_c_*	warpage
Deng et al., 2011 [21]	Kriging	*T_mold_*, *T_melt_*, *t_inj_*, *P_p_*	warpage
Xia et al., 2011 [23]	GP	*t_p_*, *p_p_*, *P_i_*, *T_melt_*, *t_inj_*, *t_c_*	warpage
Cheng et al., 2013 [24]	BPN	*t_p_*, *p_p_*, *t_c_*	warpage, *t_r_*, *t_i_*, *t_p_*, *t_c_*
Chen et al., 2014 [29]	Taguchi, PSO-GA	*T_melt_*, *t_inj_*, *t_p_*, *P_p_*, *T_c_*, *t_c_*	part length, warpage
Kitayama et al., 2014 [25]	RBF	*P_f_*	warpage
Kitayama et al. 2018 [36]	RBF, SAO	*T_mold_*, *T_melt_*, *t_inj_*, *t_p_*, *P_p_*, *T_c_*, *t_c_*	warpage, *t_c_*, weldline, *F_c_*
Zhou et al., 2021 [27]	DSFM, SVR, GPR, Kriging	*T_melt_*, *v_inj_*, *t_p_*, *P_p_*, *t_c_*	warpage, weldline, *F_c_*
This study	LHD, DNN, GA, MCS	*T_mold_*, *T_melt_*, *t_inj_*, *t_p_*, *P_p_*, *T_c_*, *t_c_*	FOT, warpage, PEEQ

Abbreviations: analysis of variance (ANOVA), back propagation neural network (BPN), constrained non-dominated sorting genetic algorithm (CNSGA), differential sensitivity fusion method (DSFM), expected improvement (EI), finite element analysis (FEA), Gaussian process (GP), Gaussian process (GP), genetic algorithm (GA), Latin hypercube design (LHD), quadratic polynomial (QP), radial basis function (RBF), response surface model (RSM), sequential approximate optimization (SAO), variable complexity methods (VCMs), injection velocity (*v_inj_*), packing profile (*p_f_*), runner type (*t_r_*), gate location (*l_g_*), mold temperature (*T_mold_*), melt temperature (*T_melt_*), injection time (*t_i_*), injection pressure (*i_p_*), quadratic polynomial (QP), cycle time (t_c_), clamping force (*F_c_*), packing time (*t_p_*), packing pressure (*P_p_*), cooling time (*t_c_*), mode-pursuing sampling (MPSs).

**Table 2:** Material properties of PA6-20CF.

Property	Value
Melt/Solid density [g/cm^3^]	0.91407/1.0177
Recommended injection temperature [°C]	285
Elastic module [GPa]	2.24
Shear module [MPa]	804.6
Poisson ratio	0.392
Maximum shear stress [MPa]	0.3
Thermal conductivity (66 °C) [W/(m∙°C)]	0.13
Specific heat (85 °C) [J/(kg∙°C)]	1756
Recommend mold temperature [°C]	50–90
Recommend melt temperature [°C]	250–295

**Table 3:** Material properties of PA6-20CF and AA 5083. Reproduced with permission from [43], Elsevier, 2019.

Material	PA6-20CF	AA 5083
Density [kg/m^3^]	1160	2600
Young’s Modulus [GPa]	15	71
Yield Strength [MPa]	157	143
Tensile Strength [MPa]	210	289
Poisson Ratio	0.42	0.33

**Table 4:** Model constants for the Ramberg–Osgood model and anisotropic Hill’48 yield function.

Parameter	Setting Range	Value
*K*	Strength coefficient	80.6 MPa
*n*	Hardening exponent	10.16
*E_m_*	Polymer matrix elastic modulus	4.25 GPa
*E_f_*	Fiber elastic modulus	105.6 GPa
*α_m_*	Weight factor of the fiber direction	2.125
*β_m_*	Weight factor of the direction normal to the fibers	1.214
*λ_m,I_*	Fiber orientation matrix 1st eigenvalue (strong fiber alignment region)	0.85

**Table 5:** Moldflow FVM simulations configurations.

Parameter	Mesh Type	Dual Domain
Mesh	Element	Type: Triangle, Number, 9620
	Aspect ratio	Maximum: 9.65 Minimum: 1.03
	Match Percentage	Mutual Percentage: 98.3%
Boundary Conditions	Valve Gate Controller	Gate Trigger: Flow front
	Cooling	Coolant: Pure water Coolant Inlet Temperature: 20 °C

**Table 6:** The ranges for parameter settings.

Control Factor	Setting Range
Melt temperature (°C)	50~90
Mold temperature (°C)	250~290
Injection time (s)	4~8
Packing pressure (MPa)	100~120
Packing time (s)	1~4
Cooling time (s)	20~120
Cooling temperature (°C)	20~60

**Table 7:** Process parameter optimization result comparison.

Case	Parameters							Objectives		
	*T*_*mold*_	*T*_*melt*_	*t*_*inj*_	*t*_*p*_	*P*_*p*_	*T*_*c*_	*t*_*c*_	FOT	Warpage	PEEQ
Best FOT case	79	288	4.6	103.1	3.8	24.5	103	0.83	16.8	0.39
Best Warpage case	65	278	7.7	108.6	1.6	58.6	42	0.52	7.8	0.47
Best PEEQ case	51	260	5.3	119.1	3.1	48.6	89	0.69	11.9	0.38
Best training case	76	265	6.4	121	3.1	51.5	82	0.52	17.8	0.38
Training average value	76.3	277.1	5.8	108	2.8	43.9	78	0.58	12.1	0.41
Optimization case	65	282	4.5	116	2.3	32.5	85	0.53	12.9	0.37
FEA case	65	282	4.5	116	2.3	32.5	85	0.52	13.1	0.38
Error (RAE)	-	-	-	-	-	-	-	1.88%	1.55%	2.27%

## Data Availability

The data presented in this study are available upon request from the corresponding author. The data are not publicly available due to privacy.

## References

[1] Choi D.S., Im Y.T. (1999). Prediction of shrinkage and warpage in consideration of residual stress in integrated simulation of injection molding. Compos. Struct..

[2] Wan Y., Takahashi J. (2016). Tensile properties and aspect ratio simulation of transversely isotropic discontinuous carbon fiber reinforced thermoplastics. Compos. Sci. Technol..

[3] Yan X., Cao S. (2018). Structure and interfacial shear strength of polypropylene-glass fiber/carbon fiber hybrid composites fabricated by direct fiber feeding injection molding. Compos. Struct..

[4] Pini T., Caimmi F., Briatico-Vangosa F., Frassine R., Rink M. (2017). Fracture initiation and propagation in unidirectional CF composites based on thermoplastic acrylic resins. Eng. Fract. Mech..

[5] Lee J., Lee H., Kim N. (2023). Fiber Orientation and Strain Rate-Dependent Tensile and Compressive Behavior of Injection Molded Polyamide-6 Reinforced with 20% Short Carbon Fiber. Polymers.

[6] Quagliato L., Kim Y., Fonseca J.H., Han D., Yun S., Lee H., Park N., Lee H., Kim N. (2020). The influence of fiber orientation and geometry-induced strain concentration on the fatigue life of short carbon fibers reinforced polyamide-6. Mater. Des..

[7] Kashyap S., Datta D. (2015). Process parameter optimization of plastic injection molding: A review. Int. J. Plast. Technol..

[8] Kitayama S. (2022). Process parameters optimization in plastic injection molding using metamodel-based optimization: A comprehensive review. Int. J. Adv. Manuf. Technol..

[9] Gaspar-Cunha A., Covas J.A., Sikora J. (2022). Optimization of Polymer Processing: A Review (Part II-Molding Technologies). Materials.

[10] Fernandes C., Pontes A.J., Viana J.C., Gaspar-Cunha A. (2018). Modeling and Optimization of the Injection-Molding Process: A Review. Adv. Polym. Technol..

[11] Ozcelik B., Sonat I. (2009). Warpage and structural analysis of thin shell plastic in the plastic injection molding. Mater. Des..

[12] Oktem H., Erzurumlu T., Uzman I. (2007). Application of Taguchi optimization technique in determining plastic injection molding process parameters for a thin-shell part. Mater. Des..

[13] Tang S.H., Tan Y.J., Sapuan S.M., Sulaiman S., Ismail N., Samin R. (2007). The use of Taguchi method in the design of plastic injection mould for reducing warpage. J. Mater. Process Technol..

[14] Kurt M., Kamber O.S., Kaynak Y., Atakok G., Girit O. (2009). Experimental investigation of plastic injection molding: Assessment of the effects of cavity pressure and mold temperature on the quality of the final products. Mater. Des..

[15] Masato D., Rathore J., Sorgato M., Carmignato S., Lucchetta G. (2017). Analysis of the shrinkage of injection-molded fiber-reinforced thin-wall parts. Mater. Des..

[16] Shi F., Lou Z.L., Lu J.G., Zhang Y.Q. (2003). Optimisation of plastic injection moulding process with soft computing. Int. J. Adv. Manuf. Technol..

[17] Kurtaran H., Ozcelik B., Erzurumlu T. (2005). Warpage optimization of a bus ceiling lamp base using neural network and genetic algorithm. J. Mater. Process Technol..

[18] Kurtaran H., Erzurumlu T. (2006). Efficient warpage optimization of thin shell plastic parts using response surface methodology and genetic algorithm. Int. J. Adv. Manuf. Technol..

[19] Ozcelik B., Erzurumlu T. (2006). Comparison of the warpage optimization in the plastic injection molding using ANOVA, neural network model and genetic algorithm. J. Mater. Process Technol..

[20] Zhang Y., Deng Y.M., Sun B.S. (2009). Injection molding warpage optimization based on a mode-pursuing sampling method. Polym.-Plast. Technol. Eng..

[21] Deng Y.M., Zhang Y., Lam Y.C. (2011). A hybrid of mode-pursuing sampling method and genetic algorithm for minimization of injection molding warpage. Mater. Des..

[22] Shi H., Gao Y., Wang X. (2010). Optimization of injection molding process parameters using integrated artificial neural network model and expected improvement function method. Int. J. Adv. Manuf. Technol..

[23] Xia W., Luo B., Liao X.P. (2011). An enhanced optimization approach based on Gaussian process surrogate model for process control in injection molding. Int. Adv. Manuf. Technol..

[24] Cheng J., Liu Z., Tan J. (2013). Multiobjective optimization of injection molding parameters based on soft computing and variable complexity method. Int. J. Adv. Manuf. Technol..

[25] Kitayama S., Onuki R., Yamazaki K. (2014). Warpage reduction with variable pressure profile in plastic injection molding via sequential approximate optimization. Int. J. Adv. Manuf. Technol..

[26] Zhao J., Cheng G., Ruan S., Li Z. (2015). Multi-objective optimization design of injection molding process parameters based on the improved efficient global optimization algorithm and non-dominated sorting-based genetic algorithm. Int. J. Adv. Manuf. Technol..

[27] Wang Y.Q., Kim J.G., Song J.I. (2014). Optimization of plastic injection molding process parameters for manufacturing a brake booster valve body. Mater. Des. (1980–2015).

[28] Shie J.R. (2008). Optimization of injection molding process for contour distortions of polypropylene composite components by a radial basis function network. Int. J. Adv. Manuf. Technol..

[29] Shen C., Wang L., Cao W. (2007). Optimization for injection molding process conditions of the refrigerator top cover using combination method of artificial neural network and genetic algorithms. PolymPlast Technol. Eng..

[30] Shen C., Wang L., Qian L. (2007). Optimization of injection molding process parameters using combination of artificial neural network and genetic algorithm method. J. Mater. Process Technol..

[31] Gao Y., Wang X. (2008). An effective warpage optimization method in injection molding based on the Kriging model. Int. J. Adv. Manuf. Technol..

[32] Gao Y., Wang X. (2009). Surrogate-based process optimization for reducing warpage in injection molding. J. Mater. Process Technol..

[33] Dimla D.E., Camilotto M., Miani F. (2005). Design and optimisation of conformal cooling channels in injection moulding tools. J. Mater. Process. Technol..

[34] Wang X., Gu J., Shen C., Wang X. (2015). Warpage optimization with dynamic injection molding technology and sequential optimization method. Int. J. Adv. Manuf. Technol..

[35] Hashimoto S., Kitayama S., Takano M., Kubo Y., Aiba S. (2020). Simultaneous optimization of variable injection velocity profile and process parameters in plastic injection molding for minimizing weldline and cycle time. J. Adv. Mech. Des. Syst. Manuf..

[36] Kitayama S., Yamazaki Y., Takano M., Aiba S. (2018). Numerical and experimental investigation of process parameters optimization in plastic injection molding using multi-criteria decision making. Simul. Model. Pract. Theory.

[37] Kitayama S., Tamada K., Takano M., Aiba S. (2018). Numerical and experimental investigation on process parameters optimization in plastic injection molding for weldlines reduction and clamping force minimization. Int. J. Adv. Manuf. Technol..

[38] Kitayama S., Tamada K., Takano M., Aiba S. (2018). Numerical optimization of process parameters in plastic injection molding for minimizing weldlines and clamping force using conformal cooling channel. J. Manuf. Process..

[39] Guo F., Jeong H., Park D., Sung B., Kim N. (2024). Numerical multi-objective optimization of segmented and variable blank holder force trajectories in deep drawing based on DNN-GA-MCS strategy. Int. J. Adv. Manuf. Technol..

[40] Guo F., Jeong H., Park D., Kim G., Sung B., Kim N. (2024). Numerical Optimization of Variable Blank Holder Force Trajectories in Stamping Process for Multi-Defect Reduction. Materials.

[41] KOPA^®^ KN111 PA6 Datasheet.

[42] Torayca^®^ T700S Datasheet.

[43] Fonseca J.H., Han G., Quagliato L., Kim Y., Choi J., Keum T., Kim S., Han D.S., Kim N., Lee H. (2019). Design and numerical evaluation of recycled-carbon-fiber-reinforced polymer/metal hybrid engine cradle concepts. Int. J. Mech. Sci..

[44] Folgar F., Tucker C.L. (1984). Orientation Behavior of Fibers in Concentrated Suspensions. J. Reinf. Plast. Compos..

[45] Quagliato L., Lee J., Fonseca J.H., Han D., Lee H., Kim N. (2021). Influences of stress triaxiality and local fiber orientation on the failure strain for injection-molded carbon fiber reinforced poly-amide-6. Eng. Fract. Mech..

[46] Bere P., Dudescu M., Neamțu C., Cocian C. (2021). Design, manufacturing and test of CFRP front hood concepts for a light-weight vehicle. Polymers.

[47] Ramesh C.K., Srikari S., Suman M.L.J. (2012). Design of hood stiffener of a sedan car for pedestrian safety. SASTech J..

[48] Darwish S.M., Elseufy S.M., Ahmad A. Finite Element Analysis of an Automobile Engine Hood. Proceedings of the 2013 International Conference on Industry, Engineering, and Management Systems.

[49] Kumar J.R., Shanmukhi K., Satyanarayana S.G., Metzler J.B. (2019). Design and Analysis of Car Hood Made with Natural Fibers. Lecture Notes on Multidisciplinary Industrial Engineering.

[50] Rodke R.R., Korade D.N. (2015). Development of Car Hood for Stiffness Improvement using FEA System. Int. J. Sci. Res. Dev..

[51] Miettinen K.M. (1998). Nonlinear Multiobjective Optimization Kluwer.

[52] Hamilton A., Tran T., Mckay M.B., Quiring B., Vassilevski P.S. (2019). Dnn Approximation of Nonlinear Finite Element Equations.

[53] Jamli M.R., Farid N.M. (2019). The sustainability of neural network applications within finite element analysis in sheet metal forming: A review. Measurement.

[54] He K., Zhang X., Ren S., Sun J. Deep residual learning for image recognition. Proceedings of the IEEE Conference on Computer Vision and Pattern Recognition.

[55] Simonyan K., Zisserman A. (2014). Very deep convolutional networks for large-scale image recognition. arXiv.

[56] Szegedy C., Liu W., Jia Y., Sermanet P., Reed S., Anguelov D., Erhan D., Vanhoucke V., Rabinovich A. Going deeper with convolutions. Proceedings of the IEEE Conference on Computer Vision and Pattern Recognition.

[57] Glorot X., Bordes A., Bengio Y. Deep sparse rectifier neural networks. Proceedings of the Fourteenth International Conference on Artificial Intelligence and Statistics.

[58] Nair V., Hinton G.E. Rectified linear units improve restricted boltzmann machines. Proceedings of the 27th international conference on machine learning (ICML-10).

[59] Cui B., Guo H., Zhou Z.H. Multi-task deep neural networks for non-linear regression. Proceedings of the Thirtieth AAAI Conference on Artificial Intelligence (AAAI).

[60] Zhang W., Wu X., Liu T. (2018). A comparative study of deep neural networks for non-linear regression. J. Comput. Sci. Technol..

[61] Huang M.C., Tai C.C. (2001). The effective factors in the warpage problem of an injection-molded part with a thin shell feature. J. Mater. Process Technol..

[62] Erzurumlu T., Ozcelik B. (2006). Minimization of warpage and sink index in injection-molded thermoplastic parts using Taguchi optimization method. Mater. Des..

[63] Chen W.C., Kurniawan D. (2014). Process parameters optimization for multiple quality characteristics in plastic injection molding using Taguch method, BPNN, GA, and hybrid PSO-GA. Int. J. Precis. Eng. Manuf..

